# Proinflammatory cytokines induce accumulation of glypican-1-derived heparan sulfate and the C-terminal fragment of β-cleaved APP in autophagosomes of dividing neuronal cells

**DOI:** 10.1093/glycob/cwaa011

**Published:** 2020-02-10

**Authors:** Fang Cheng, Lars-Åke Fransson, Katrin Mani

**Affiliations:** Department of Experimental Medical Science, Division of Neuroscience, Glycobiology Group, Lund University, 221 00 Lund, Sweden

**Keywords:** Alzheimer’s disease, amyloid precursor protein, glypican-1, heparan sulfate, inflammatory cytokines

## Abstract

Proinflammatory cytokines stimulate expression of β-secretase, which increases processing of amyloid precursor protein (APP), ultimately leading to the deposition of amyloid beta (Aβ). The N-terminal domain of β-cleaved APP supports Cu/NO-dependent release of heparan sulfate (HS) from the glypican-1 (Gpc-1) proteoglycan. HS is an inhibitor of β-secretase, thereby constituting a regulatory, negative feedback loop. Here, we have investigated the effect of the proinflammatory cytokines TNF-α, IL-1β and IL-6 on the interplay between APP processing and release of HS from Gpc-1 in neuronal cells. We have used deconvolution immunofluorescence microscopy and sodium dodecyl sulfate polyacrylamide gel electrophoresis (SDS-PAGE) and a panel of monoclonal/polyclonal antibodies recognizing the released HS, the N-terminus of Aβ, Aβ, the C-terminus of APP and the autophagosome marker LC3 as well as the chemical lysosome marker LysoTrackerRed (LTR). We repeatedly found that N2a neuroblastoma cells and human neural stem cells grown in the presence of the cytokines developed large cytoplasmic clusters, which stained positive for HS, the N-terminus of Aβ, Aβ, the C-terminus of APP, LC3 and LTR, indicating accumulation of HS and APP/APP degradation products in enlarged autophagosomes/lysosomes. The SDS-PAGE of immunoisolates obtained from TNF-α-treated N2a cells by using anti-C-terminus of APP revealed the presence of SDS-stable complexes between HS and the C-terminal fragment of β-cleaved APP (βCTF) migrating in the range 10–18 kDa. Clustered accumulation of βCTF disappeared when HS release was prevented and slightly enhanced when HS release was increased. Hence, when proinflammatory cytokines induce increased processing of APP, inhibition of β-secretase by HS is insufficient, which may lead to the impaired autophagosomal degradation.

## Introduction

Neuroinflammation is believed to contribute to neurodegeneration in Alzheimer’s disease (AD). Increased levels of proinflammatory cytokines, such as tumor necrosis factor-α (TNF-α), interleukin-1β (IL-1β) and interleukin-6 (IL-6), have been found in serum and brain tissue of AD patients. These cytokines can be derived from activated microglia or enter the brain via a leaky blood–brain barrier during a systemic inflammation. The cytokines stimulate expression of β-secretase, thereby increasing the processing of amyloid precursor protein (APP), ultimately leading to deposition of amyloid beta (Aβ) (for recent reviews, see Morris et al. 2014; Heneka et al. 2015; Calsolaro and Edison 2016; Wang et al. 2017).

**Fig. 1:**
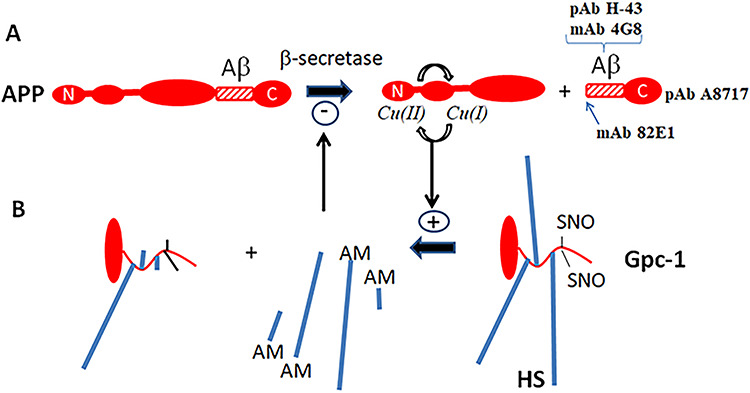
Regulation of amyloid precursor protein (APP) and Gpc-1 processing. APP is cleaved by β-secretase that yields a large N-terminal and a small C-terminal fragment (**A**, left-to-right). The antibodies used to identify the C-terminal fragment are indicated. γ-Secretase then releases Aβ from the C-terminal fragment (not shown). The N-terminal fragment of APP catalyzes, via a copper redox cycle, the deaminative release of HS chains and oligosaccharides from Gpc-1 (**B**, right-to-left, +). The released heparan sulfate (HS) inhibits β-secretase (**A**, −). N, N-terminus; C, C-terminus; AM, anMan; SNO, S-nitrosothiol. It should be noted that several deaminative cleavage sites are clustered at the proximal end of the HS chain, giving rise both long and short fragments. This figure is available in black and white in print and in color at *Glycobiology* online.

There is a mutual functional relationship between APP and the recycling heparan sulfate (HS)-containing proteoglycan glypican-1 (Gpc-1). These two membrane-bound proteins interact strongly with one another and co-localize in the endosomes (Cappai et al. 2005; Cheng et al. 2014, 2019). There, APP is cleaved by β- and γ-secretases (Figure 1A; for reviews, see Reinhard et al. 2005; O’Brien and Wong 2011). The N-terminal fragment of β-cleaved APP (βNTF) contains binding sites for copper ions and HS (Reinhard et al. 2005). βNTF supports copper-dependent release of HS from Gpc-1 by S-nitrosothiol (SNO)-catalyzed deaminative cleavage, generating anhydromannose-containing HS chains and oligosaccharides (HS-anMan) (Figure 1A and B, +; see also Cheng et al. 2012, 2017a). The release of HS-anMan can be spontaneous or induced by ascorbate. Both are completely abolished in APP^−/−^ fibroblasts but can be restored by transfection with a vector encoding APP. Likewise, HS release is greatly suppressed when β-secretase is inhibited both in mouse fibroblasts and N2a neuroblastoma cells (Cheng et al. 2014). In fibroblasts from an AD mouse model (Tg2576, carrying the APP-swe mutation), there is increased β-cleavage of APP and accumulation of Aβ. Accordingly, the rate of HS-anMan formation is also increased in these cells (Cheng et al. 2015).

HS is an inhibitor of β-secretase/BACE1 (Scholefield et al. 2003), thereby constituting a regulatory, negative feedback loop (Figure 1A, −). Thus, silencing of Gpc-1 expression in Tg2576 fibroblasts enhances Aβ accumulation (Cheng et al. 2011). Dividing N2a neuroblastoma cells and human neural stem cells (NSCs) exposed to the cyanobacterial neurotoxin β-N-methylamino-L-alanine (BMAA) generates HS-deficient Gpc-1 and displays increased APP processing (Cheng et al. 2019). SNO-catalyzed release of HS requires constant NO production. Accordingly, NO-deprivation results in increased APP processing in mouse fibroblasts and N2a cells (Cheng et al. 2015, 2019).

Here, we have investigated the effect of TNF-α, IL-1β and IL-6 on the interplay between APP processing and release of HS from Gpc-1 in neuronal cells. We repeatedly found that the cytokines induced formation of complexes between HS-anMan and APP degradation products, which accumulated in enlarged autophagosomes/lysosomes of dividing mouse N2a neuroblastoma and human neuronal stem cells (NSCs). This may contribute to autophagosomal dysfunction in AD.

## Results

### Proinflammatory cytokines induce accumulation of HS-anMan and APP/APP degradation products in enlarged autophagosomes/lysosomes of growing N2a neuroblastoma cells

**Fig. 2:**
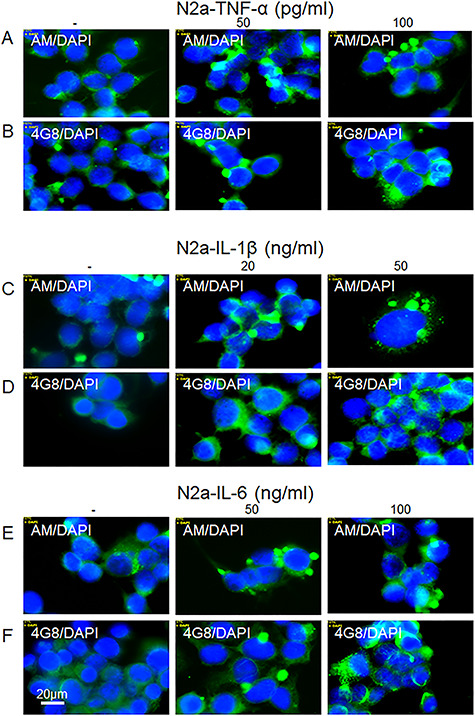
Proinflammatory cytokines induce accumulation of HS-anMan and Aβ immunoreactivity in cytoplasmic clusters of growing mouse N2a neuroblastoma cells. Representative immunofluorescence images of cells that were grown to near confluence for 48 h in regular medium (−) or in medium containing the indicated concentrations of tumor necrosis factor-α (TNF-α) (**A**, **B**), IL-1β (**C**, **D**) or IL-6 (**E**, **F**). Staining was performed with mAb AM (for HS-anMan, green), 4G8 (for Aβ, green) and DAPI (for nuclei, blue). Exposure time was the same in all cases. Bar, 20 μm. It should be noted that N2a cells tend to grow in clusters, which can be more or less pronounced. This figure is available in black and white in print and in color at *Glycobiology* online.

We examined the effects of the cytokines by deconvolution immunofluorescence microscopy using mAbs and pAbs recognizing the released HS-anMan, the N-terminus of Aβ, Aβ, the C-terminus of APP and the autophagosome marker LC3 (Figure 1). Mouse N2a neuroblastoma cells spontaneously generated HS-anMan, which was detected in the cytoplasm using mAb AM (Figure 2A, C and E; see untreated, −). When cells were grown to confluence in the presence of increasing concentrations of the cytokines TNF-α, IL1-β or IL-6, a distinct qualitative change was repeatedly observed. Staining for HS-anMan was increasingly concentrated to large cytoplasmic clusters (Figure 2A, C and E; cf. untreated with the indicated concentrations). Similar results were obtained when cells were stained for Aβ by using mAb 4G8 (Figure 2B, D and F; see the indicated concentrations).

**Fig. 3:**
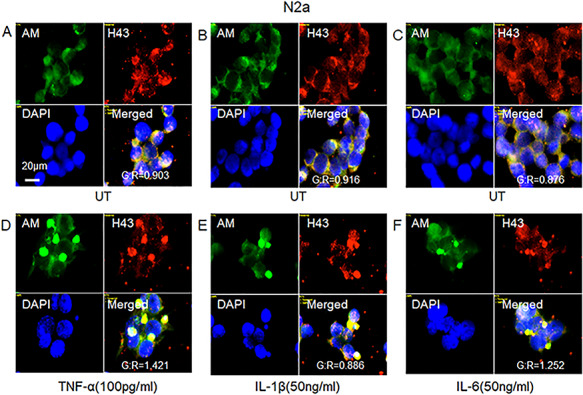
HS-anMan and Aβ immunoreactivity co-localize in cytoplasmic clusters induced by proinflammatory cytokines in growing mouse N2a neuroblastoma cells. Representative immunofluorescence images of cells that were grown to near confluence for 48 h in regular medium (**A**–**C**; UT = untreated) or in medium containing the indicated concentrations of TNF-α (**D**), IL-1β (**E**) or IL-6 (**F**). Staining was performed with mAb AM (for HS-anMan), pAb H-43 (for Aβ) and DAPI (for nuclei). Exposure time was the same in all cases. Bar, 20 μm. The extent of co-localization is expressed as the G/R ratio (green vs. red channel). This figure is available in black and white in print and in color at *Glycobiology* online.

**Fig. 4:**
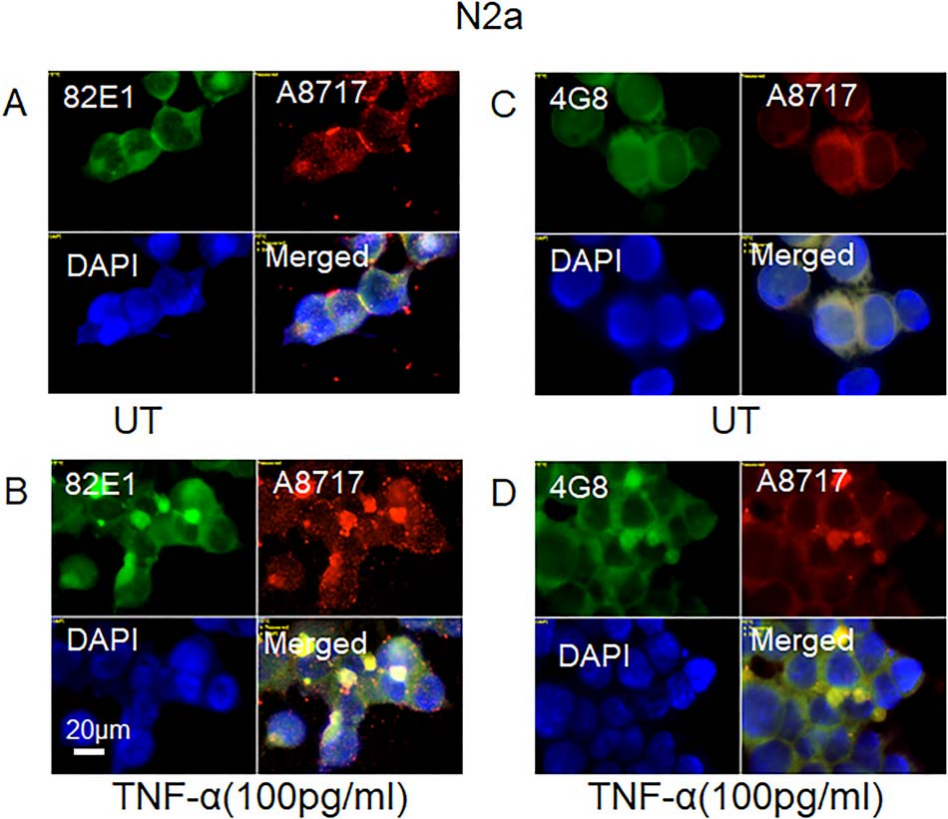
Staining for the N-terminus of Aβ, Aβ and the C-terminus of APP co-localize in cytoplasmic clusters induced by TNF-α in growing mouse N2a neuroblastoma cells. Representative immunofluorescence images of cells that were grown to near confluence for 48 h in a regular medium (**A**, **C**; UT = untreated) or in medium containing TNF-α (**B**, **D**; 100 pg/ml). Staining was performed with mAb 82E1 (for the N-terminus of β-cleaved C-terminal fragment of APP), mAb 4G8 (for Aβ), pAb A8717 (for the C-terminus of APP) and DAPI (for nuclei). Exposure time was the same in all cases. Bar, 20 μm. This figure is available in black and white in print and in color at *Glycobiology* online.

**Fig. 5:**
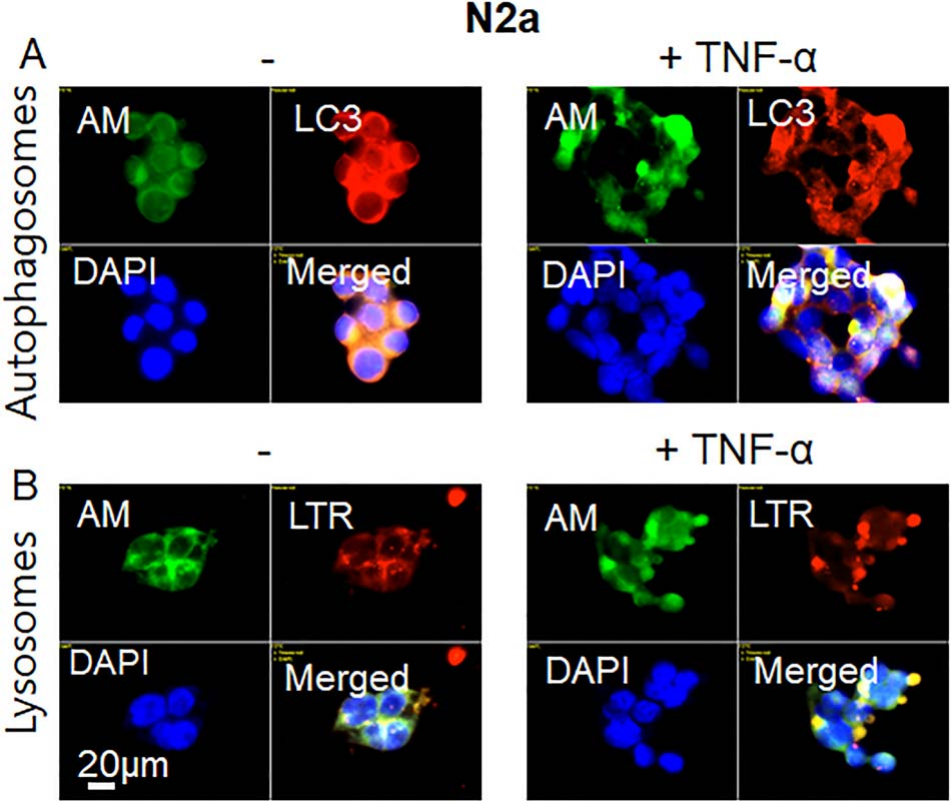
The TNF-α-induced complexes between APP-β-CTF and HS-anMan are located in enlarged autophagosomes/lysosomes of growing mouse N2a neuroblastoma cells. Representative immunofluorescence images of cells that were grown to near confluence for 48 h in regular medium (**A**, **B**, −) or in medium containing 100 pg/ml TNF-α (**A**, **B**, TNF-α). Staining was performed with mAb AM (for HS-anMan), pAb anti-LC3 (for autophagosomes), LTR (for lysosomes) and DAPI (for nuclei). Exposure time was the same in all cases. Bar, 20 μm. This figure is available in black and white in print and in color at *Glycobiology* online.

To examine if HS-anMan and APP/APP degradation products co-localized in the cytoplasmic clusters, we co-stained for HS-anMan with mAb AM and for Aβ content with pAb H-43. In untreated N2a cells, there was diffuse co-localization of HS-anMan and Aβ in the cytoplasm (Figure 3A–C, yellow in merged; G/R ~0.9), whereas co-localization was concentrated to the cytoplasmic clusters induced by the indicated concentrations of the three cytokines (Figure 3D–F, yellow in merged; G/R 0.9–1.4).

As the cytokines stimulate expression of β-secretase, we assessed the occurrence of β-cleavage of APP by using a mAb specific for the N-terminus of Aβ (82E1; see Figure 1). Staining with 82E1 was observed in the cytoplasm of untreated N2a cells indicating that β-cleavage of APP had taken place (Figure 4A). There was also diffuse co-localization with staining for the C-terminus of APP (A8717) (Figure 4A, merged). In contrast, co-localization of the two epitopes was concentrated to the cytoplasmic clusters generated during growth in the presence of TNF-α (Figure 4B, merged; cf. Figure 4A, merged). Co-staining with mAb 4G8 (for the Aβ segment) and pAb A8717 (for the C-terminus of APP) also showed co-localization in the cytoplasmic clusters of TNF-α-treated cells and diffuse staining in the untreated cultures (cf. Figure 4C and D, merged).

To identify the location of the cytoplasmic clusters containing HS-anMan and the APP degradation products, we co-stained untreated and TNF-α-treated N2a cells with mAb AM (for HS-anMan) and pAb anti-LC3 (for autophagosomes) or LysoTracker Red (for lysosomes). TNF-α induced clustered co-localization of both HS-anMan and LC3 (Figure 5A, −/+ TNF-α, yellow in merged) and of HS-anMan and LTR (Figure 5B, −/+ TNF-α, yellow in merged). Hence, HS-anMan and APP degradation products appear to accumulate in enlarged autophagosomes/lysosomes.

### TNF-α induces formation of SDS-stable complexes between APP-βCTF and HS-anMan in growing N2a neuroblastoma cells

**Fig. 6:**
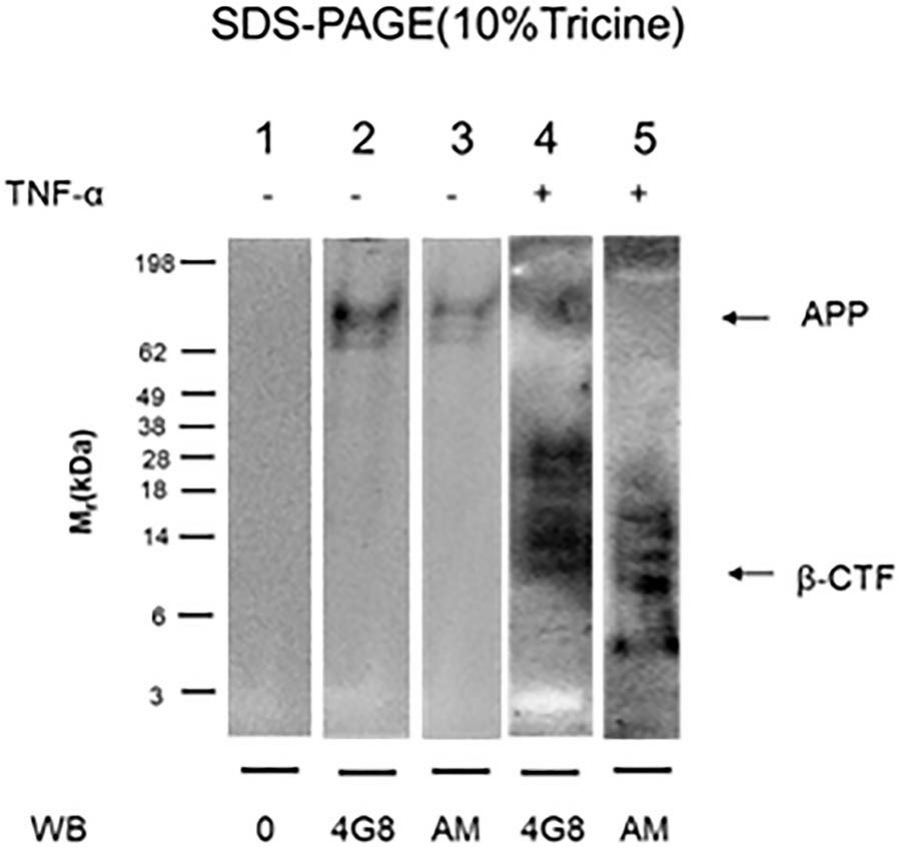
TNF-α induces formation of SDS-stable complexes between APP-βCTF and HS-anMan in growing mouse N2a neuroblastoma cells. The cells were either untreated (−) or grown for 48 h in the presence of 100 pg/ml of TNF-α (+). Immunoprecipitation (IP) was performed on the cell extracts with pAb A8717, which recognizes the C-terminus of APP. The same amount of protein was applied to each lane, and electrophoresis was followed by transfer to PVDF membranes and western blotting using either mAb 4G8 (for Aβ) or mAb AM (for HS-anMan). In lane 1, the primary antibody was omitted (0). A band corresponding to APP as in lane 4 was also seen when IP was performed with pAb Aβ40 (not shown).

To distinguish between APP and the APP degradation products generated during growth of N2a cells in the absence or presence of TNF-α, we performed SDS-PAGE and western blotting. Cells were lysed, nuclei were spun down and supernatants were immunoprecipitated by using pAb A8717, which recognizes the C-terminus of APP. The immunoprecipitates were recovered by Dynabeads coated with anti-rabbit IgG and a magnet concentrator. The immunoisolate obtained from untreated cells yielded a 4G8-positive band corresponding to APP, which was also stained by mAb AM, but no βCTF (Figure 6, lanes 2 and 3). Apparently, spontaneously generated HS-anMan oligosaccharides were associated with APP.

**Fig. 7:**
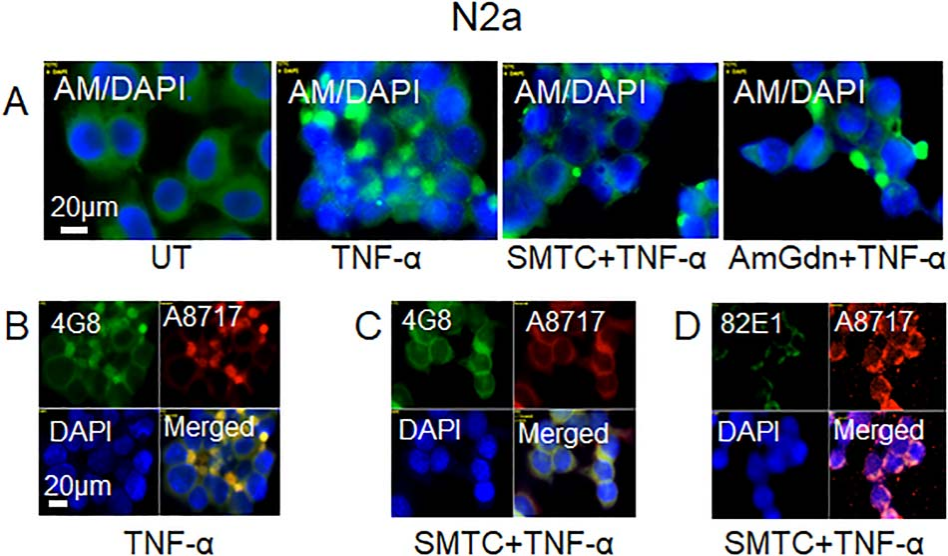
Inhibition of HS-anMan release by NO-deprivation prevents TNF-α-induced clustering of HS-anMan and APP/APP degradation products in growing mouse N2a neuroblastoma cells. Representative immunofluorescence images of cells that were grown to near confluence for 48 h in regular medium (UT = untreated) or in medium containing TNF-α (100 pg/ml), SMTC (100 μM), aminoguanidine (AmGdn, 10 mM) or combinations thereof as indicated below the images. Staining was performed with mAb AM (for HS-anMan), mAb 4G8 (for Aβ), mAb 82E1 (for the N-terminus of Aβ), pAb A8717 (for the C-terminus of APP) and DAPI (for nuclei). Exposure time was the same in all cases. Bar, 20 μm. This figure is available in black and white in print and in color at *Glycobiology* online.

**Fig. 8:**
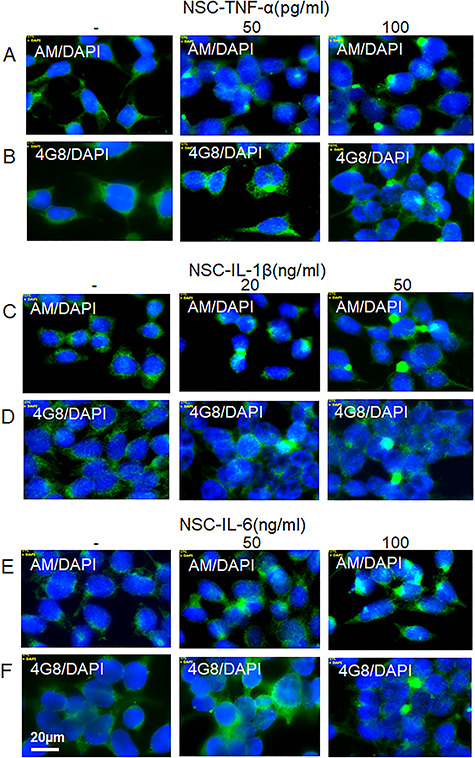
Proinflammatory cytokines induce accumulation of HS-anMan and Aβ immunoreactivity in cytoplasmic clusters of growing human neural stem cells (NSCs). Representative immunofluorescence images of cells that were grown to near confluence for 48 h in regular medium (−) or in medium containing the indicated concentrations of TNF-α (**A**, **B**), IL-1β (**C**, **D**) or IL-6 (**E**, **F**). Staining was performed with mAb AM (for HS-anMan, green), 4G8 (for Aβ, green) and DAPI (for nuclei, blue). Exposure time was the same in all cases. Bar, 20 μm. This figure is available in black and white in print and in color at *Glycobiology* online.

SDS-PAGE of the A8717 immunoisolate obtained from TNF-α-treated N2a cells showed a weak band in the position of APP and several 4G8-positive components ranging in size from ~10 to 40 kDa (Figure 6, lane 4). The shorter ones (10–18 kDa) also stained positive for HS-anMan (Figure 6, lane 5). Thus, when APP processing was augmented in TNF-α-treated cells, there was accumulation of SDS-stable complexes between APP-βCTF and HS-anMan.

### Inhibition of HS-anMan release by NO-deprivation prevents TNF-α-induced clustering of HS-anMan and APP/APP degradation products in growing N2a neuroblastoma cells

As deaminative release of HS-anMan from the Gpc-1 proteoglycan requires prior S-nitrosylation of the Gpc-1 protein (Cheng et al. 2011, 2012), suppression of NO production should result in decreased capacity to generate HS-anMan. N2a cells express neuronal NO-synthase (nNOS), which can be selectively inhibited by S-methyl-L-thiocitrulline (SMTC) as shown previously (Cheng et al. 2019). The TNF-α-induced clustering of HS-anMan in growing N2a cells (Figure 7A, cf. UT = untreated and TNF-α) was less prominent after simultaneous treatment with SMTC (Figure 7A, cf. TNF-α and SMTC + TNF-α). Aminoguanidine, which inhibits inducible NO-synthase (iNOS), was not that effective (Figure 7A, cf. TNF-α and AmGdn + TNF-α). Cultures treated with TNF-α alone or with SMTC were stained with mAb A8717 (for the C-terminus of APP) and either 4G8 (for Aβ) or 82E1 (for the N-terminus of Aβ) as indicated. There was no clustered co-localization of Aβ and the C-terminus of APP when cells were grown in the presence of both SMTC and TNF-α (cf. Figure 7B and C, merged). Moreover, staining for the N-terminus of Aβ was weak (Figure 7D, 82E1) suggesting a low content of APP-βCTF.

**Fig. 9:**
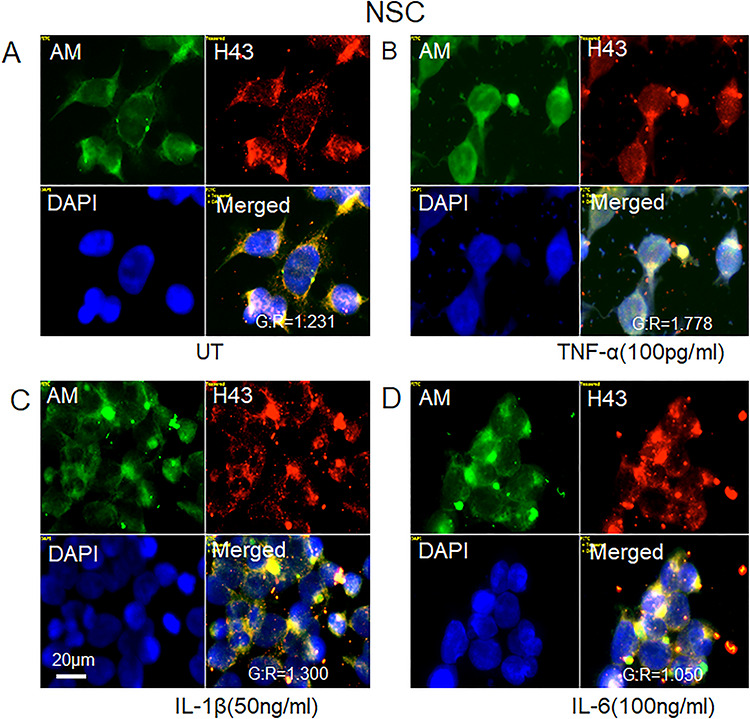
HS-anMan and Aβ immunoreactivity co-localize in the cytoplasmic clusters induced by proinflammatory cytokines in growing human NSCs. Representative immunofluorescence images of cells that were grown to near confluence for 48 h in regular medium (**A**; UT = untreated) or in medium containing the indicated concentrations of TNF-α (**B**), IL-1β (**C**) or IL-6 (**D**). Staining was performed with mAb AM (for HS-anMan), pAb H-43 (for Aβ) and DAPI (for nuclei). Exposure time was the same in all cases. Bar, 20 μm. The extent of co-localization is expressed as the G/R ratio (green versus red channel). This figure is available in black and white in print and in color at *Glycobiology* online.

### Proinflammatory cytokines induce NO-dependent, cytoplasmic clustering of HS-anMan and APP/APP degradation products in enlarged autophagosomes/lysosomes of growing human NSCs

We also examined the effect of the proinflammatory cytokines on HS-anMan release and APP processing in human NSC by deconvolution immunofluorescence microscopy. Cells grown to confluence in regular medium spontaneously generated HS-anMan (Figure 8A, C and E, −, AM). When grown in the presence of the cytokines TNF-α, IL1-β or IL-6, staining for HS-anMan became increasingly concentrated to cytoplasmic clusters (Figure 8A, C and E, AM; see the indicated concentrations). Similar results were obtained for Aβ (Figure 8B, D and F, 4G8; see the indicated concentrations).

To show that HS-anMan and Aβ co-localized in the cytoplasmic clusters, we co-stained for HS-anMan (mAb AM) and Aβ (pAb H-43). In untreated NSC, there was diffuse co-localization of HS-anMan and Aβ in the cytoplasm (Figure 9A, yellow in merged; G/R 1.2) but clustered co-localization in cells exposed to the cytokines (Figure 9B–D, yellow in merged; G/R 1.1–1.8).

**Fig. 10:**
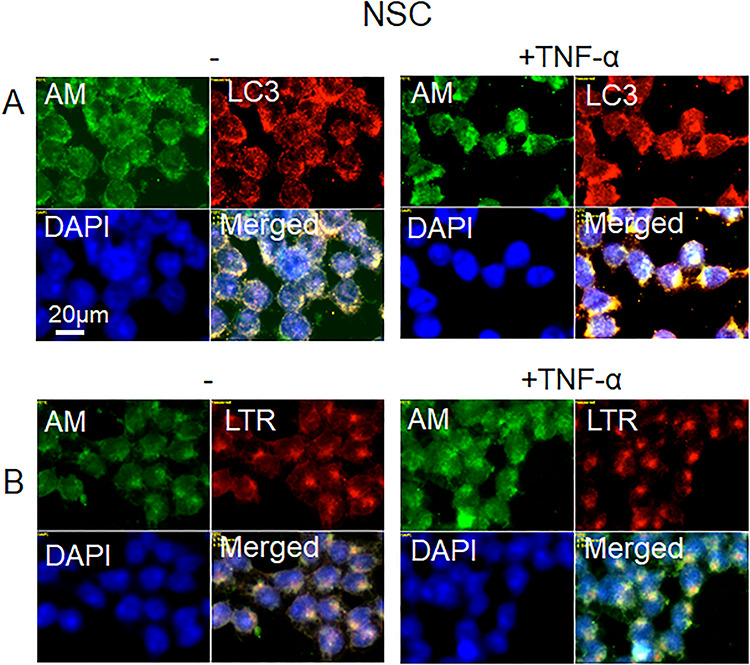
TNF-α induces accumulation of HS-anMan in enlarged autophagosomes/lysosomes of growing human NSCs. Representative immunofluorescence images of cells that were grown to near confluence for 48 h in regular medium (**A**, **B**, −) or in medium containing 100 pg/ml TNF-α (**A**, **B**, TNF-α). Staining was performed with mAb AM (for HS-anMan), pAb anti-LC3 (for autophagosomes), LTR (for lysosomes) and DAPI (for nuclei). Exposure time was the same in all cases. Bar, 20 μm. This figure is available in black and white in print and in color at *Glycobiology* online.

To identify the location of the HS-anMan-containing clusters, we co-stained untreated and TNF-α-treated NSC with mAb AM (for HS-anMan) and pAb anti-LC3 (for autophagosomes) or LysoTracker Red (for lysosomes). In the TNF-α-induced clusters of HS-anMan staining (Figure 10A and B; −/+ TNF-α, AM), there was co-localization with LC3 (Figure 10A, +TNF-α, AM, LC3 and yellow in merged) and to some extent with LTR (Figure 10B, TNF-α, AM and merged). Hence, in NSC, HS-anMan appeared to accumulate primarily in enlarged autophagosomes.

**Fig. 11:**
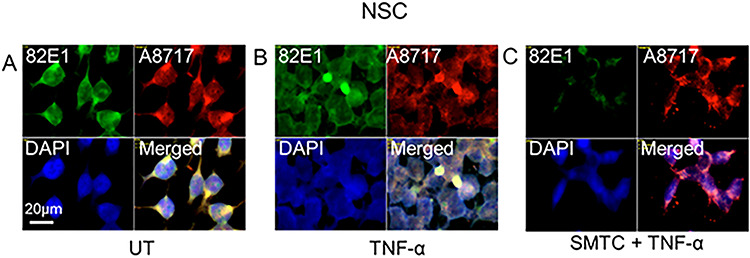
TNF-α induces HS-dependent formation of cytoplasmic clusters containing β-cleaved APP in growing human NSCs. Representative immunofluorescence images of cells that were grown to near confluence for 48 h in regular medium (**A**; UT = untreated) or in medium containing TNF-α (**B**; 100 pg/ml) or 100 μM SMTC + TNF-α (**C**). Staining was performed with mAb 82E1 (for the N-terminus of β-cleaved APP), pAb A8717 (for the C-terminus of APP) and DAPI (for nuclei). Exposure time was the same in all cases. Bar, 20 μm. This figure is available in black and white in print and in color at *Glycobiology* online.

To assess the extent of β-cleavage, we co-stained NSC with mAb 82E1 (for the N-terminus of Aβ) and pAb A8717 (for the C-terminus of APP). In untreated cells, there was mostly diffuse co-localization of the two epitopes (Figure 11A, yellow in merged), whereas TNF-α induced increased clustered co-localization (Figure 11B, merged). Hence, cytokine-exposed NSC also appeared to accumulate β-cleaved APP in cytoplasmic clusters. When HS-anMan formation was suppressed by using the NO-synthase inhibitor SMTC in combination with TNF-α, staining for the N-terminus of β-cleaved APP was weak and no clustering was observed (Figure 11C, 82E1 and merged).

### Stimulation of HS-anMan release by ascorbate slightly enhances TNF-α-dependent accumulation of HS-anMan and APP degradation products in enlarged cytoplasmic vesicles of growing neural cells

Spontaneous generation of HS-anMan is maintained by a copper redox cycle in APP-βNTF (Figure 1A). Exogenously supplied ascorbate can also induce release of HS-anMan. We have previously provided evidence for an electron transfer chain involving uptake of ascorbate into the cytosol, oxidation and transfer of electrons across the endosomal membrane via cytochrome b561 to APP-βNTF leading to increased regeneration of Cu(I) (Cheng et al. 2017a). Therefore, to increase HS-anMan formation, N2a and NSC cells were grown to confluence in the presence of ascorbate or both TNF-α and ascorbate.

**Fig. 12:**
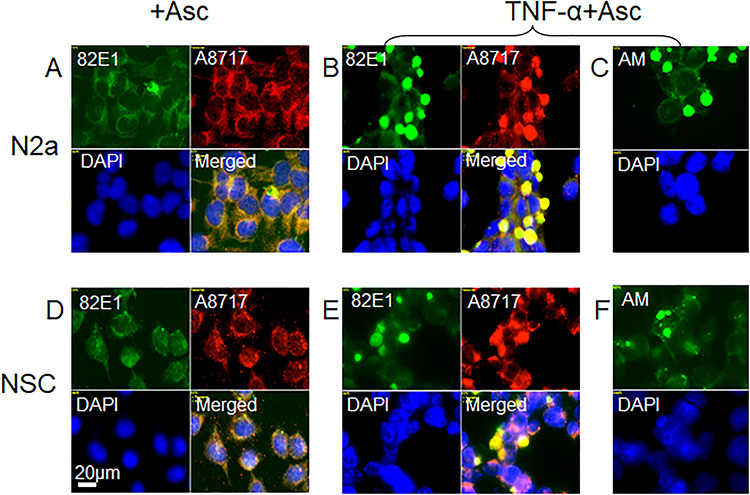
Stimulation of HS-anMan release by ascorbate slightly enhances TNF-α-dependent accumulation of HS-anMan and APP degradation products in enlarged cytoplasmic vesicles of growing neural cells. Representative immunofluorescence images of N2a neuroblastoma cells (**A–C**) or human NSCs (**D–F**) that were grown to near confluence for 48 h in medium containing 1 mM ascorbate (Asc; **A**, **D**) or 100 pg/ml of TNF-α and 1 mM of ascorbate (TNF-α + Asc; **B**, **C**, **E**, **F**). Staining was performed with mAb 82E1 (for the N-terminus of β-cleaved APP), pAb A8717 (for the C-terminus of APP), mAb AM (for HS-anMan) and DAPI (for nuclei). Exposure time was the same in all cases. Bar, 20 μm. This figure is available in black and white in print and in color at *Glycobiology* online.

When grown in the presence of ascorbate alone, neither N2a nor NSC cells developed clustered staining with mAb 82E1 (for the N-terminus of βCTF) or pAb A8717 (for the C-terminus of βCTF) (Figure 12A and D). However, when grown in the presence of both TNF-α and ascorbate, intense co-localization of the two epitopes appeared in a slightly increased number of enlarged cytoplasmic vesicles (Figure 12B and E). Also HS-anMan accumulated in these vesicles (Figure 12C and F).

### No cytokine-induced clustering of HS-anMan and no increased β-cleavage of APP in proinflammatory cytokine-treated primary cultures of nondividing mouse cortical neurons

**Fig. 13:**
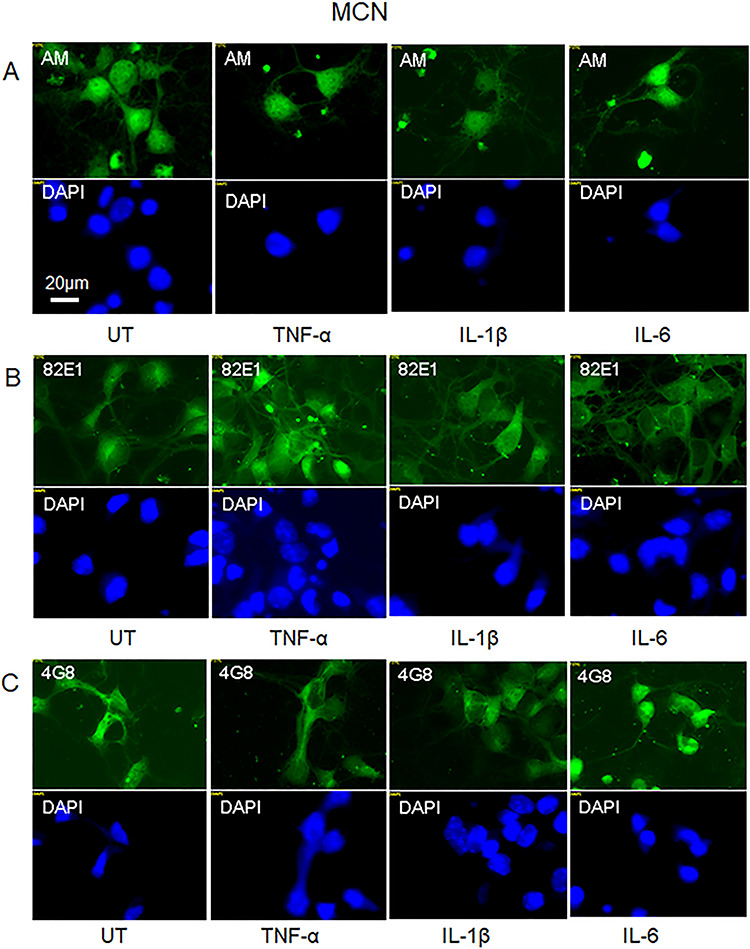
No effect on HS-anMan formation and β-cleavage of APP by proinflammatory cytokines in primary cultures of nondividing mouse cortical neurons (MCNs). Representative immunofluorescence images of cells that were kept for 48 h in regular medium (**A–C**, UT = untreated) or in medium containing TNF-α (**A–C**, 100 pg/ml), IL-1β (**A–C**, 50 ng/ml) or IL-6 (**A–C**, 100 ng/ml). Staining was performed with mAb AM (for HS-anMan), mAb 82E1 (for the N-terminus of the β-cleaved C-terminal fragment of APP), mAb 4G8 (for Aβ) and DAPI (for nuclei). Cells were fixed in acetone (**A**) or paraformaldehyde (**B**, **C**). Exposure time was the same in all cases. Bar, 20 μm. This figure is available in black and white in print and in color at *Glycobiology* online.

Primary cultures of nondividing mouse cortical neurons (MCNs) generated HS-anMan, which appeared both in the nuclei and in the cytoplasm, including the neurites (Figure 13A, AM, UT = untreated). Exposure to the cytokines TNF-α, IL-1β, or IL-6 had no effect on the intracellular distribution of HS-anMan (Figure 13A, AM; see the indicated cytokines). In untreated neurons, staining for the N-terminus of β-cleaved APP appeared in the cytoplasm (Figure 13B, 82E1, UT). No consistent change in the distribution of this staining was observed in the cytokine-treated neurons (Figure13B, 82E1; see the indicated cytokines). Similarly, staining for Aβ appeared in the cytoplasm of untreated neurons (Figure 13C, 4G8, UT) and remained mostly unaffected by the cytokines (Figure 13C, 4G8; see the indicated cytokines).

## Discussion

**Fig. 14:**
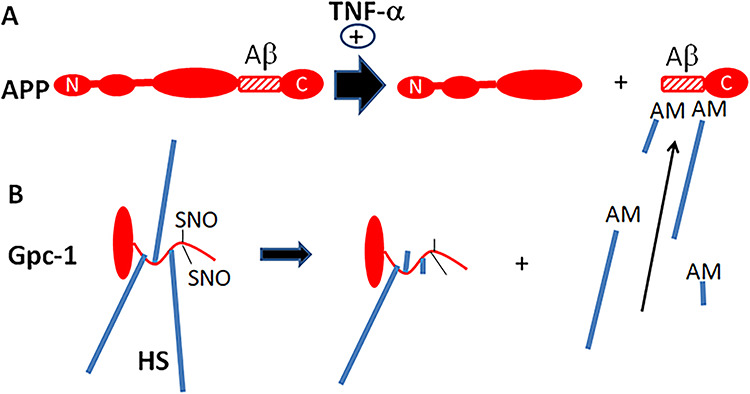
Cytokine-induced formation of complexes between HS-anMan and APP βCTF. TNF-α induces increased β-cleavage of APP, thereby raising the levels of both βNTF and βCTF (**A**). Spontaneous SNO-catalyzed processing of Gpc-1 generates HS-anMan that is captured by βCTF (**B**). If most of the HS-anMan is bound to βCTF, inhibition of β-secretase by HS should be minimal. This figure is available in black and white in print and in color at *Glycobiology* online.

In untreated cells, only a portion of APP was processed by β-cleavage and no accumulation of βCTF was observed. Apparently, a balance between βNTF-promoted release of HS-anMan and inhibition of β-secretase (BACE1) by HS-anMan was established (Figure 1). This balance can be disturbed either by increased degradation of APP or reduced release of HS-anMan from Gpc-1. Proinflammatory cytokines greatly increased the rate of β-cleavage of APP, which in turn should enhance HS-anMan release from Gpc-1. However, if most of the HS-anMan became bound to βCTF, there would be insufficient inhibition of β-secretase. A continued high rate of β-cleavage of APP would then ensue resulting in accumulation of complexes between βCTF and HS-anMan in enlarged autophagosomes/lysosomes (Figure 14).

Degradation of these complexes by HS-degrading enzymes and by γ-secretase may be very slow. We have previously demonstrated the presence of SDS-stable complexes between APP-βCTF and HS-anMan in mouse Tg2576 fibroblasts. These complexes were resistant to HS-degrading enzymes and partly resistant to proteinase K (Cheng et al. 2011). There is also impaired lysosomal degradation of HS and Aβ in mouse Tg2576 fibroblasts (Cheng et al. 2015). Intraneuronal accumulation of APP-βCTF associated with autophagosome/lysosome dysfunction has been observed in familial AD and in mouse models of AD (Lauritzen et al. 2016; Kaur et al. 2017; Hung and Livesey 2018). In Down syndrome (Trisomi-21) there is increased expression of APP due to an extra copy of the APP gene. In fibroblasts from individuals with Down syndrome and in cortical neurons from mouse models of this syndrome, there is lysosomal dysfunction, which is mediated by the elevated levels of βCTF (Ying et al. 2019).

The generation of βCTF as well as the release of HS-anMan from Gpc-1 takes place in the endosomes. HS-anMan then penetrates the endosomal membrane and is transported to the nucleus and then to autophagosomes/lysosomes. In mouse fibroblasts and N2a neuroblastoma cells, β-cleavage of APP is required both for HS-anMan formation and for egress from the endosomes. Passage through the membrane requires generation of the membrane-bound βCTF (Cheng et al. 2014, 2017a). When there is overproduction of βCTF, complexes between βCTF and HS-anMan may exit into the cytosol and be captured in autophagosomes resulting in autophagosome/lysosome dysfunction.

Reduced release of HS-anMan should result in increased β-cleavage of APP (Figure 1). When HS-deficient Gpc-1 was generated by exposure to the cyanobacterial neurotoxin N-methylamino-L-alanine (BMAA), there was an increased APP processing in both mouse N2a and human NSCs and accumulation of β-cleaved APP degradation products in cytoplasmic clusters. Similar results were obtained by NO-deprivation, which prevents HS-anMan release (Cheng et al. 2019). In the present study, when N2a cells were grown in the presence of both TNF-α and SMTC (NOS-inhibitor), no accumulation of HS-anMan and APP degradation products was observed. In this case, reduced release of HS-anMan in the context of greatly increased APP processing may have resulted in unimpeded degradation of βCTF and Aβ byautophagy.

Stimulation of HS-anMan release by ascorbate slightly increased cluster formation of HS and APP degradation products in TNF-α-treated mouse N2a cells and in human NSCs. If the capacity to generate HS-anMan was almost fully utilized during the spontaneous process in untreated cells, there should be little further release of HS-anMan when the cytokine-induced level of βNTF was increased and when cells were simultaneously treated with ascorbate. Hence, inhibition of β-secretase by HS would still be insufficient.

NO is required for the generation of HS-anMan. Transgenic AD mice with deletions of either the inducible or the endothelial NOS genes display an enhanced AD-like pathology (Colton et al. 2008; Hu et al. 2013). In fibroblasts from individuals with Niemann–Pick type C1 disease, there is reduced generation of HS-anMan and impeded egress from the endosomes to the cytosol (Cheng et al. 2017a). In this disease, defective endosomal transport of cholesterol and neuropathological lesions typical of AD have been detected (Jin et al. 2004).

Past and present results thus indicate that HS chains, released by an NO-dependent mechanism from the Gpc-1 proteoglycan, are important modulators/regulators of APP processing and amyloid formation. However, when proinflammatory cytokines induce greatly increased processing of APP, the regulatory function of HS is insufficient.

## Materials and methods

### Cells and reagents

Mouse N2a neuroblastoma cells were the same as used previously (Cheng et al. 2014). MCNs were purchased from Fisher Scientific, plated in polylysine-coated dishes and maintained as recommended by the supplier. Human NSCs were obtained from Applied Stem Cell Inc. Matrigel, growth factors and stem cell dissociating reagent (Accutase) were from ATCC. Cells were grown in DMEM (Life Technologies) and maintained as described in the attached protocols. TNF-α was from Alomone labs, IL-1β from Gibco and IL-6 from Tataa Biocenter). mAb AM was a monoclonal antibody recognizing anhydromannose (anMan)-containing HS; mAb 4G8 was used to detect Aβ and pAb 8717 to detect the C-terminus of APP as described previously (Cheng et al. 2014, 2015, 2017a,b). A mAb specific for the N-terminus of Aβ (82E1) was from IBL. pAbs against Aβ were from Santa Cruz (H-43) or Thermo Fisher (Aβ40, PA3–16760), anti-LC3 from Sigma-Aldrich, sheep anti-rabbit IgG-coated Dynabeads M-280 from In Vitrogen, fluorescein isothiocyanate (FITC)-labeled goat anti-mouse Ig from Sigma-Aldrich and Alexa Fluor 594-labeled goat or donkey anti-rabbit IgG from Life Technologies. Antibodies were used for immunofluorescence microscopy, for western blots and, in some cases, for immunoisolation as recommended by the manufacturers. The DNA staining compound 4,6-diaminido-2-phenylindole (DAPI), BMAA, L-serine, aminoguanidine and ascorbate were from Sigma-Aldrich and LysoTrackerRed from Molecular Probes. SMTC was purchased from Santa Cruz. A proteinase inhibitor kit (cOmplete, mini) was from Roche.

### Deconvolution immunofluorescence microscopy

Cells were examined by immunofluorescence microscopy as described previously (Cheng et al. 2014). In brief, cells were fixed in acetone in order to retain cellular and subcellular structures and to ensure the preservation of carbohydrates. Fixation in paraformaldehyde was used in two cases as indicated. The fixed cells were first pre-coated with 10% anti-mouse total Ig and then exposed to primary antibodies overnight. The secondary antibodies used were FITC-tagged goat anti-mouse Ig when the primary antibody was a monoclonal and Alexa Fluor 594-tagged goat anti-rabbit IgG or sometimes Alexa Fluor 594-tagged donkey anti-goat IgG when the primary antibody was a polyclonal. In the controls, the primary antibody was omitted. DNA staining with 4,6-diamidino-2-phenylindole (DAPI), as well as staining with antibodies, was performed as recommended by the manufacturers. The fluorescent images were analyzed by using a Carl Zeiss Axio Observer inverted fluorescence microscope with deconvolution technique and equipped with objective EC “Plan-Neofluar” 63x/1.25 Oil M27 and AxioCam MRm Rev Camera. Identical exposure settings and times were used for all images. During microscopy, the entire slides were scanned, and representative immunofluorescence images containing many cells were captured. For co-localization analysis, the fluorophores were excited in a sequential manner using multitrack acquisition to minimize cross talk. Data analysis was performed using Zeiss AxioVision Release 4.8 software. Entire images were used for estimation of the extent of co-localization.

### Immunoisolation and SDS-PAGE

APP/ΑPP-degradation products were immunoisolated from cell extracts by using a pAb against the C-terminus of APP (A8717). Cells were lysed and nuclei were removed from the lysate by centrifugation using a nucleus/cytosol separation kit as described previously (Cheng et al. 2014). The supernatant was mixed with 0.1 vols. of 10X RIPA buffer, i.e., 1% (w/v) SDS, 5% (v/v) Triton X-100 and 5% (w/v) sodium deoxycholate in PBS and supplemented with proteinase inhibitors (cOmplete mini) and 0.5 mM phenylmethylsulfonyl fluoride at 4°C. Antibody was added, and immune complexes were recovered by using Dynabeads coated with anti-rabbit IgG and a magnetic particle concentrator. Immunoisolates were displaced by using SDS, and SDS-PAGE was performed on 10% Tricine gels followed by transfer to polyvinylidene fluoride (PVDF) membranes, which were then probed with mAb 4G8 or mAb AM and visualized, all as described in detail previously (Cheng et al. 2011). Protein was measured by using the BCA assay kit from Pierce.

## Abbreviations

Aβ: amyloid beta
AD: Alzheimer’s disease
anMan/AM: anhydromannose
APP: amyloid precursor protein
APP-βCTF: C-terminal fragment of β-cleaved APP (C99)
APP-γCTF: C-terminal fragment of γ-cleaved APP
APP-βNTF: N-terminal fragment of β-cleaved APP
BACE1: beta-site APP cleaving enzyme (β-secretase)
BMAA: β-N-methylamino-L-alanine
DAPI: 4,6-diaminido-2-phenylindole
FITC: fluorescein isothiocyanate
Gpc-1: glypican-1
HS: heparan sulfate
iNOS: inducible nitric oxide synthase
IL-1β: interleukin-1β
IL-6: interleukin-6
LTR: lysoTracker Red
mAb: monoclonal antibody
MCN: mouse cortical neuron
NO: nitric oxide
nNOS: neuronal nitric oxide synthase
NSC: neural stem cell
pAb: polyclonal antibody
PVDF: polyvinylidene fluoride
SMTC: S-methyl-L-thiocitrulline
SNO: S-nitrosothiol
Tg2576: transgenic for the Swedish mutation of APP
TNF-α: tumor necrosis factor-α

## References

[ref1] Calsolaro V, Edison P 2016 Neuroinflammation in Alzheimer’s disease: Current evidence and future directions. Alzheimers Dement. 12:719–732. 27179961 10.1016/j.jalz.2016.02.010

[ref2] Cappai R, Cheng F, Ciccotosto GD, Needham BE, Masters CL, Multhaup G, Fransson L-Å, Mani K 2005 The amyloid precursor protein (APP) of Alzheimer disease and its paralog, APLP2, modulate the cu/Zn-nitric oxide-catalyzed degradation of glypican-1 heparan sulfate *in vivo*. J Biol Chem. 280:13913–13920. 15677459 10.1074/jbc.M409179200

[ref3] Cheng F, Cappai R, Ciccotosto GD, Svensson G, Multhaup G, Fransson L-Å, Mani K 2011 Suppression of amyloid β A11 antibody immunoreactivity by vitamin C. possible role of heparan sulfate oligosaccharides derived from glypican-1 by ascorbate-induced, nitric oxide (NO)-catalyzed degradation. J Biol Chem. 286:27559–27572. 21642435 10.1074/jbc.M111.243345 PMC3149348

[ref4] Cheng F, Svensson G, Fransson L-Å, Mani K 2012 Non-conserved, S-nitrosylated cysteines in glypican-1 react with N-unsubstituted glucosamines in heparan sulfate and catalyze deaminative cleavage. Glycobiology. 22:1480–1486. 22801553 10.1093/glycob/cws111

[ref5] Cheng F, Cappai R, Lidfelt J, Belting M, Fransson L-Å, Mani K 2014 Amyloid precursor protein (APP)/APP-like protein 2 (APLP2) expression is required to initiate endosome-nucleus-autophagosome trafficking of glypican-1-derived heparan sulfate. J Biol Chem. 289:20871–20878. 24898256 10.1074/jbc.M114.552810 PMC4110294

[ref6] Cheng F, Fransson L-Å, Mani K 2015 Rapid nuclear transit and impaired degradation of amyloid β and glypican-1-derived heparan sulfate in Tg2576 mouse fibroblasts. Glycobiology. 25:548–556. 25527428 10.1093/glycob/cwu185

[ref7] Cheng F, Fransson L-Å, Mani K 2017a Cytochrome b561, copper, β-cleaved amyloid precursor protein and Niemann-pick C1 protein are involved in ascorbate-induced release and membrane penetration of heparan sulfate from endosomal S-nitrosylated glypican-1. Exp Cell Res. 360:171–179. 28893506 10.1016/j.yexcr.2017.09.003

[ref8] Cheng F, Belting M, Fransson L-Å, Mani K 2017b Nucleolin is a nuclear target for heparan sulfate derived from glypican-1. Exp Cell Res. 354:31–39. 28300561 10.1016/j.yexcr.2017.03.021

[ref9] Cheng F, Fransson L-Å, Mani K 2019 The cyanobacterial neurotoxin β-N-methylamino-L-alanine prevents addition of heparan sulfate to glypican-1 and increases processing of amyloid precursor protein in dividing neuronal cells. Exp Cell Res. 379:172–181. 30953622 10.1016/j.yexcr.2019.03.041

[ref10] Colton CA, Wilcock DM, Wink DA, Davis J, Van Nostrand WE, Vitek MP 2008 The effects of NOS2 gene deletion on mice expressing mutated human AbetaPP. J Alzheimers Dis. 15:571–587. 19096157 10.3233/jad-2008-15405 PMC2667339

[ref11] Heneka MT, Carson MJ, El Khoury J et al. 2015 Neuroinflammation in Alzheimer’s disease. Lancet Neurol. 14:388–405. 25792098 10.1016/S1474-4422(15)70016-5 PMC5909703

[ref12] Hu ZI, Kotarba AM, Van Nostrand WE 2013 Absence of nitric oxide synthase 3 increases amyloid β-protein pathology in Tg-5xFAD mice. Neurosci Med. 4:84–91. 24159423 10.4236/nm.2013.42013 PMC3804905

[ref13] Hung COY, Livesey FJ 2018 Altered gamma-secretase processing of APP disrupts lysosome and autophagosome function in monogenic Alzheimer’s disease. Cell Rep. 25:3647–3660 e3642. 30590039 10.1016/j.celrep.2018.11.095 PMC6315085

[ref14] Jin LW, Maezawa I, Vincent I, Bird T 2004 Intracellular accumulation of amyloidogenic fragments of amyloid-β precursor protein in neurons with Niemann-pick type C defects is associated with endosomal abnormalities. Amer J Pathol. 164:975–985. 14982851 10.1016/s0002-9440(10)63185-9 PMC1614713

[ref15] Kaur G, Pawlik M, Gandy SE, Ehrlich ME, Smiley JF, Levi E 2017 Lysosomal dysfunction in the brain of a mouse model with intraneuronal accumulation of carboxy terminal fragments of the amyloid precursor protein. Mol Psychiatry. 22:981–989. 27777419 10.1038/mp.2016.189 PMC5405008

[ref16] Lauritzen I, Pardossi-Piquard R, Bourgeois A, Pagnotta S, Biferi MG, Barkats M, Lacor P, Klein W, Bauer C, Checler F 2016 Intraneuronal aggregation of the beta-CTF fragment of APP (C99) induces Abeta-independent lysosomal-autophagic pathology. Acta Neuropathol. 132:257–276. 27138984 10.1007/s00401-016-1577-6 PMC4947121

[ref17] Morris JK, Honea RA, Vidoni ED, Swerdlow RH, Burns JM 2014 Is Alzheimer’s disease a systemic disease? Biochim Biophys Acta. 1842:1340–1349. 24747741 10.1016/j.bbadis.2014.04.012 PMC4126236

[ref18] O’Brien RJ, Wong PC 2011 Amyloid protein processing and Alzheimer’s disease. Annu Rev Neurosci. 34:185–204. 21456963 10.1146/annurev-neuro-061010-113613 PMC3174086

[ref19] Reinhard C, Hébert SS, De Strooper B 2005 The amyloid-β precursor protein: Integrating structure with biological function. EMBO J. 24:3996–4006. 16252002 10.1038/sj.emboj.7600860 PMC1356301

[ref20] Scholefield Z, Yates EA, Wayne G, Amour A, McDowell W, Turnbull JE 2003 Heparan sulfate regulates amyloid precursor protein processing by BACE1, the Alzheimer’s beta-secretase. J Cell Biol. 163:97–107. 14530380 10.1083/jcb.200303059 PMC2173449

[ref21] Wang J, Gu BJ, Masters CL, Wang Y-J 2017 A systemic view of Alzheimer disease-insights from amyloid-β metabolism beyond the brain. Nat Rev Neurol. 13:612–623. 28960209 10.1038/nrneurol.2017.111

[ref22] Ying J, Sato Y, Im E, Berg M, Bordi M, Darji S, Kumar A, Mohan PS, Bandyopadhyay U, Diaz A et al. 2019 Lysosomal dysfunction in down syndrome is APP-dependent and mediated by APP-βCTF (C99). J Neurosci. 39:5255–5268. 31043483 10.1523/JNEUROSCI.0578-19.2019 PMC6607756

